# Maternal epigenetic signatures are associated with small for gestational age births among black women

**DOI:** 10.48130/epi-0026-0006

**Published:** 2026-07-30

**Authors:** Paolo Reho, Tingting Zhao, Yihong Zhao, Goleen Samari, Ronald Wapner, Arielle Hazi, Haotian Wu, Veronica Barcelona

**Affiliations:** 1 Department of Environmental Health Sciences, Mailman School of Public Health, Columbia University, New York, NY 10032, USA; 2 School of Nursing, Columbia University, New York, NY 10032, USA; 3 Population and Public Health Sciences, Keck School of Medicine, University of Southern California, Los Angeles, CA 90033, USA; 4 Department of Obstetrics and Gynecology at Columbia University Irving Medical Center, New York, NY 10032, USA

**Keywords:** Small for gestational age, Epigenome-wide association study, DNA methylation, Epigenomics, Biomarker, Black women, Pregnancy

## Abstract

Approximately 10% US infants are born small for gestational age (SGA), a condition linked to increased morbidity and mortality. Infants born to Black women are twice as likely to be SGA compared with those born to White women. Although maternal factors, including epigenetic modifications, likely contribute to SGA, the underlying biological mechanisms remain poorly understood. We evaluated whether epigenetic modifications in early pregnancy were associated with SGA among pregnant Black women. We analyzed data from 931 pregnant non-Hispanic Black women (6–13 weeks of gestation) enrolled in the Nulliparous Pregnancy Outcomes Study: Monitoring Mothers-to-be (nuMoM2b) cohort across eight academic medical centers. We conducted an epigenome-wide association study using the Infinium MethylationEPIC assay on blood samples from women who delivered SGA infants (*n* = 133) and appropriate for gestational age infants (*n* = 798). We adjusted for maternal age, prenatal smoking, education, body mass index, and infant sex, and corrected with multiple testing. We identified 14 differentially methylated 5'-C-phosphate-G-3' (CpG) sites mapping to genes involved in placental development, vascular remodeling, and fetal growth regulation. Functional enrichment analysis highlighted the pathways involved in early embryonic development and placental function, implicating early-pregnancy maternal epigenetic alterations in SGA delivery among Black women and supporting DNA methylation profiling as a potential SGA biomarker.

## Introduction

Small-for-gestational-age (SGA) refers to infants whose birth weight falls below the 10th percentile for their gestational age, representing approximately 8%–10% of all live births in the United States^[1,2]^. Infants born to Black women are more than twice as likely to be born SGA compared with infants born to White women^[3]^. SGA is associated with increased risks of morbidity and mortality beginning in the neonatal period and extending into adulthood^[4-6].^ Understanding the biological mechanisms underlying SGA is therefore critical to identifying its etiologic risk factors and informing prevention and intervention strategies.

Although maternal genetic, epigenetic, and environmental factors likely play a role in the risk of SGA, the underlying biological mechanisms, particularly the role of maternal DNA methylation (DNAm) alterations, remain poorly understood. Emerging evidence suggests that the interaction between maternal DNAm and environmental exposures, especially significant maternal stress and adverse social determinants of health, can lead to epigenetic modifications^[7,8]^. Altered DNAm patterns in the genes involved in placental function, immune regulation, and growth signaling pathways (e.g., *IGF2*, *H19*, *LEP*) may disrupt fetal development and increase the risk of SGA at delivery^[9]^. Postnatally, both unmethylated and methylated placental *SERPINB5* levels have been shown to be positively associated with gestational age at birth^[10]^. However, this association is no longer evident in later infancy (beyond 11 weeks of corrected age). Given that SGA infants are at an increased risk for preterm birth, the epigenetic profiles observed in preterm populations may offer insight into shared biological pathways, although our current study only focused on the epigenetic characteristics of SGA. For example, 5'-C-phosphate-G-3' (CpG) sites mapped to genes such as *PLA2G4EM*, *TRIM9*, *GRIK3*, and *MACROD2* have been implicated in neurodevelopment and may represent common targets of interest^[11]^. Nevertheless, DNAm variations associated with maternal stress among pregnant Black women remain largely unexplored.

To address these knowledge gaps, we conducted a secondary analysis of a longitudinal cohort study of nulliparous pregnant Black women in the United States between 2010 and 2015. We analyzed DNA extracted from early-pregnancy maternal blood samples and performed an epigenome-wide association study (EWAS) to identify epigenetic changes associated with the delivery of SGA infants. The findings from this study may advance our understanding of the early biological pathways underlying fetal growth restrictions and informing future public health strategies and maternal–child health policies.

## Materials and methods

### Study cohort

The parent Nulliparous Pregnancy Outcomes Study: Monitoring Mothers-to-be (nuMoM2b) was a prospective, longitudinal study that enrolled nulliparous pregnant women between 6 weeks (6 + 0/7) and 13 weeks (13 + 6/7) of gestation. Recruitment occurred between 2010 and 2014 across eight academic medical centers: the University of California, Irvine; the University of Utah, Northwestern University, Indiana University, The Ohio State University, the University of Pittsburgh, the University of Pennsylvania, and Columbia University. Informed consent, either verbal or written, was obtained from all participants as part of the original nuMoM2b study protocol^[12]^. Our study was approved by the Institutional Review Board at Columbia University (#AAAU0215). Inclusion criteria were as follows: (1) Nulliparous pregnant women with singleton pregnancies, and (2) gestational age between 6 weeks 0 days (6 + 0/7) and 13 weeks 6 days (13 + 6/7) at the time of first-trimester enrollment. Participants' exclusion criteria included: (1) Maternal age younger than 13 years, (2) the presence of fetal malformations, (3) fetal aneuploidy, (4) gestational age less than 20 weeks, and (5) planned pregnancy termination. The study included 10,037 women, with a mean age of 26.9 years (standard deviation, SD = 5.7). Participants were predominantly non-Hispanic white (*n* = 5,988; 59.7%), followed by non-Hispanic Black (14.2%; *n* = 1,420), Hispanic (16.9%; *n* = 1,699), Asian (4.0%; *n* = 406), and other races/ethnicities (5.1%; *n* = 514). Participants were followed through three standardized study visits during pregnancy, and a fourth visit at delivery. Survey data were collected for participants, including demographic, behavioral and clinical measures, and birth outcomes were abstracted from the electronic health records after delivery.

For the present study, we conducted analyses of all eligible Black participants (*n* = 1,073) who provided DNA in the parent study and gave consent for future genetic analyses. Maternal demographic information, including age, body mass index (BMI), educational attainment, and smoking status, as well as the infants' sex, weight, and gestational age were obtained via electronic health record abstraction. We calculated SGA status (< 10^th^ percentile) using Oken criteria^[13]^.

### Genomic DNA extraction and processing

Peripheral whole blood samples were obtained from participants during their initial study visit. Genomic DNA was isolated and stored at −80 °C until future processing. Each DNA sample was bisulfite-converted using the EZ-96 DNA Methylation Kit (Zymo Research) and processed on the Infinium MethylationEPIC v2.0 BeadChips (Illumina) at the University of Minnesota Genomics Center.

### DNA methylation profiling, data quality control, and preprocessing

Raw intensity data processing and downstream statistical analysis were performed in R (v.4.0.5). Methylation data were preprocessed using the *MethylCallR* package, which provides a standardized and EPICv2-aware pipeline for quality control and CpG filtering^[14]^. Raw IDAT files were imported and quality-filtered on the basis of multiple criteria. Samples were flagged as low-quality and excluded from further analysis if (1) the median log_2_ signal intensity was below 10.5, following the default threshold implemented in the *minfi* package^[15]^, or (2) more than 1% of the CpGs exhibited a detection *p*-value greater than 0.01.

CpGs were excluded from the analysis if they met any of the following criteria: (1) detection *p*-value > 0.01 in > 1% of samples, (2) bead count < 3 in > 5% of samples, (3) mapped to a non-CG site, (4) located on sex chromosomes, (5) associated with single-nucleotide polymorphisms in the reference population (African ancestry), (6) flagged as cross-reactive or low-reproducibility CpGs^[16]^, (7) failed to map to the GRCh38 human genome reference, or (8) were EPICv2-specific replicated CpGs.

After filtering, 121,890 CpGs with poor quality and three samples with suboptimal overall quality were removed. Outlier detection based on the Mahalanobis distance was applied to identify samples with aberrant methylation profiles; 11 samples were flagged as potential outliers and excluded from further analyses. An additional 128 participants were excluded because of missing covariate and/or outcome data. The final analytic dataset included 931 samples and 815,166 high-confidence CpGs for downstream analyses. Data normalization was performed using the Noob (normal–exponential out-of-band) method with an offset of 100, without XY CpG interpolation. To minimize technical confounding, known batch variables, including Sentrix barcodes and sample sections, were adjusted using the *MeCall.RemoveBatch* function while preserving the phenotype of interest (SGA) during correction. Subsequently, the cell type composition was estimated using *MeCall.CellComp*, yielding proportions of CD8+ T, CD4+ T, natural killer (NK), and B cells, monocytes, and neutrophils. CpG gene annotation was retrieved using the *annotatr* R package using *annotate_regions* function (Supplementary Table S1)^[17]^.

### Differential methylation analysis in SGA

We performed a hypothesis-free EWAS without preselecting candidate genes or pathways. Normalized and batch-adjusted data were converted into beta values for downstream analysis. An EWAS was then performed using the *MeCall.DMP* function (*MethylCallR*), which implements a linear modeling framework based on the *limma* package^[18]^. The primary exposure of interest was infants' SGA status. To control for technical variation and potential confounding, regression models were adjusted for relevant maternal and infant covariates, including maternal age, prenatal smoking status, educational attainment, BMI at the first prenatal visit, infants' sex, estimated leukocyte cell type proportions (CD8+ T cells, CD4+ T cells, NK cells, B cells, monocytes, and neutrophils), and the first 10 principal components (derived from the methylation data). Study participants with missing covariate information were excluded from the study. We performed the EWAS using beta values, which represent the proportion of methylated CpGs, and computed the delta beta (Δ*β*) for each site. We identified differentially methylated probes (DMPs) using empirical Bayes moderated *t*-statistics, and multiple testing correction was applied using the Benjamini–Hochberg procedure to control the false discovery rate (FDR).

### Functional enrichment analysis

Functional enrichment analysis was conducted using the g:Profiler toolkit (version 0.2.3)^[19]^ to identify overrepresented biological pathways across multiple databases, including Gene Ontology (GO)^[20]^, Kyoto Encyclopedia of Genes and Genomes (KEGG)^[21]^, and Reactome^[22]^. The analysis included genes linked to DMPs that met a FDR threshold of < 0.1 in the EWAS. No candidate genes or gene sets were preselected; the analysis was performed in a hypothesis-free manner across all CpGs passing quality control, and used a custom reference background derived from the CpGs (*n* = 815,166) available in the study. To account for multiple testing, Bonferroni correction was applied, and only pathways with an adjusted *p*-value < 0.05 were retained for interpretation.

## Results

### Demographic characteristics of the study population

The study included 931 participants who self-identified as Non-Hispanic Black, of whom 133 (14.3%) delivered SGA infants and 798 (85.7%) delivered appropriate-for-gestational-age (AGA) infants. The mean maternal age at delivery was 23.5 years (SD = 5.3). The majority of the study participants had a college level education (50.4%), followed by high school or less (42.6%), whereas only a small fraction had an education level beyond college (7.0%). The mean BMI was 28.9 (SD = 7.9). There were no statistically significant differences in maternal age, education level, or BMI between the SGA and AGA groups. However, women who delivered SGA infants had a higher rates of smoking during pregnancy (16.5% vs. 9.6%; *p* = 0.017). SGA infants were also more likely to be female (61.7% vs. 47.4%). The demographic and clinical characteristics of the study population are summarized in Table 1.

### Epigenome-wide association study

**Fig. 1:**
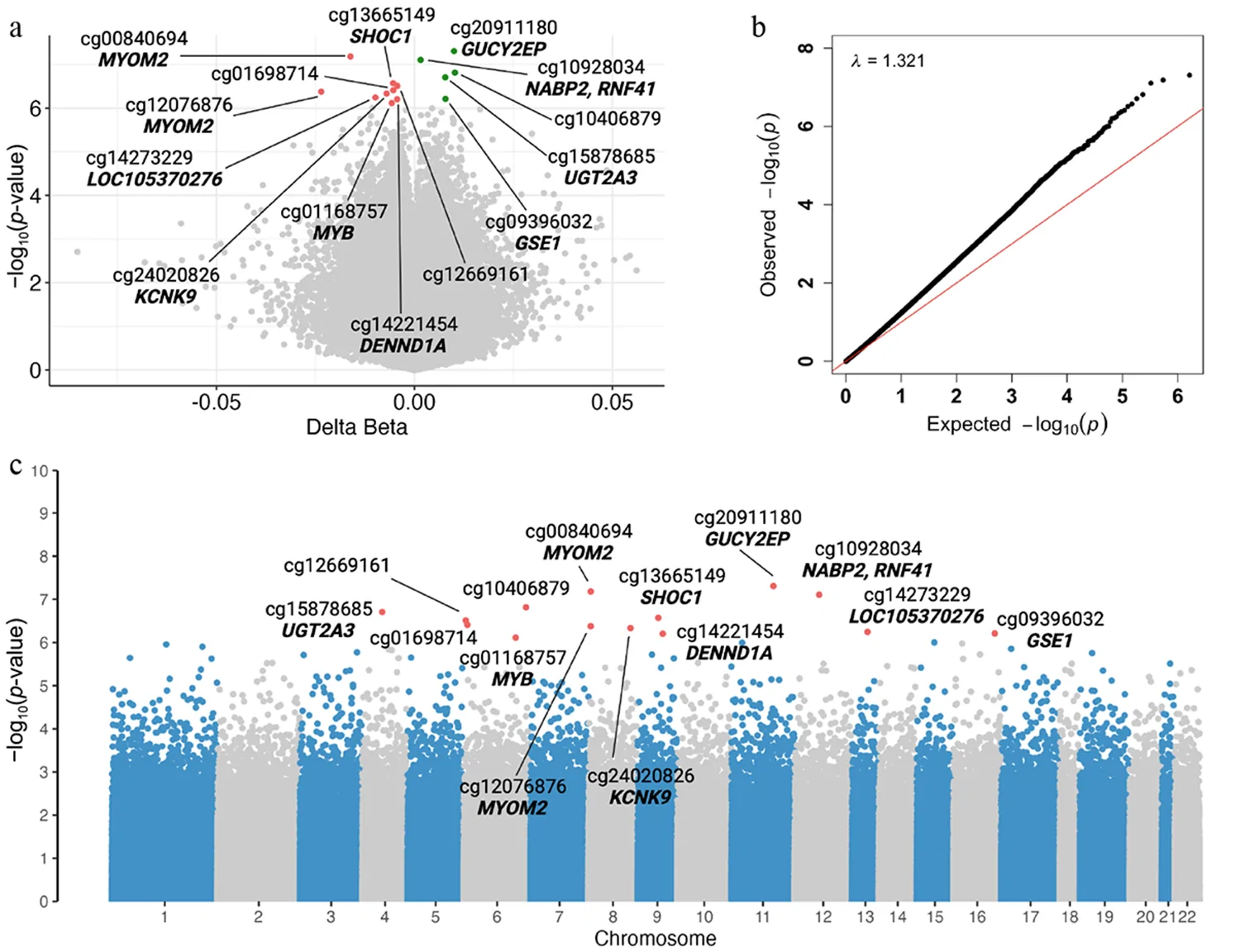
Maternal differentially methylated CpGs associated with SGA. Volcano plot (a) showing hypomethylated (left) and hypermethylated (right) DMPs. CpG sites surpassing the FDR threshold are depicted in red or green, whereas nonsignificant CpGs appear in gray. QQ plot (b) illustrating the distribution of *p*-values and the calculated genomic inflation factor (*λ*). Manhattan plot (c) displaying the genomic position of each DMP, with statistically significant sites highlighted in red. Gene and CpG annotations are shown.

We assessed the association between DNA methylation levels at individual CpG sites and SGA status. This analysis revealed 14 DMPs significantly associated with SGA after FDR correction (Table 2, Fig. 1, Supplementary Table S1).

Differences in methylation ranged from Δ*β* = −0.024 to Δ*β* = 0.010, with nine CpGs (64%) showing hypomethylation and five (36%) showing hypermethylation in SGA individuals compared with the controls. Ten of these CpGs were annotated to genes including *GUCY2EP*, *MYOM2*, *NABP2*, *RNF41*, *UGT2A3*, *SHOC1*, *KCNK9*, *LOC105370276*, *GSE1*, and *DENND1A*. Notably, two CpGs, cg00840694 (Δ*β* = −0.016; adjusted *p* = 0.021) and cg12076876 (Δ*β* = −0.024; adjusted *p* = 0.038), mapped to Intron 1 of *MYOM2* and were separated by less than 200 bp, lying within or directly upstream (5 bp) of a candidate *cis*-regulatory element (cCRE; encyclopedia of DNA elements [ENCODE] accession EH38E2606814).

### Functional enrichment analysis

**Fig. 2:**
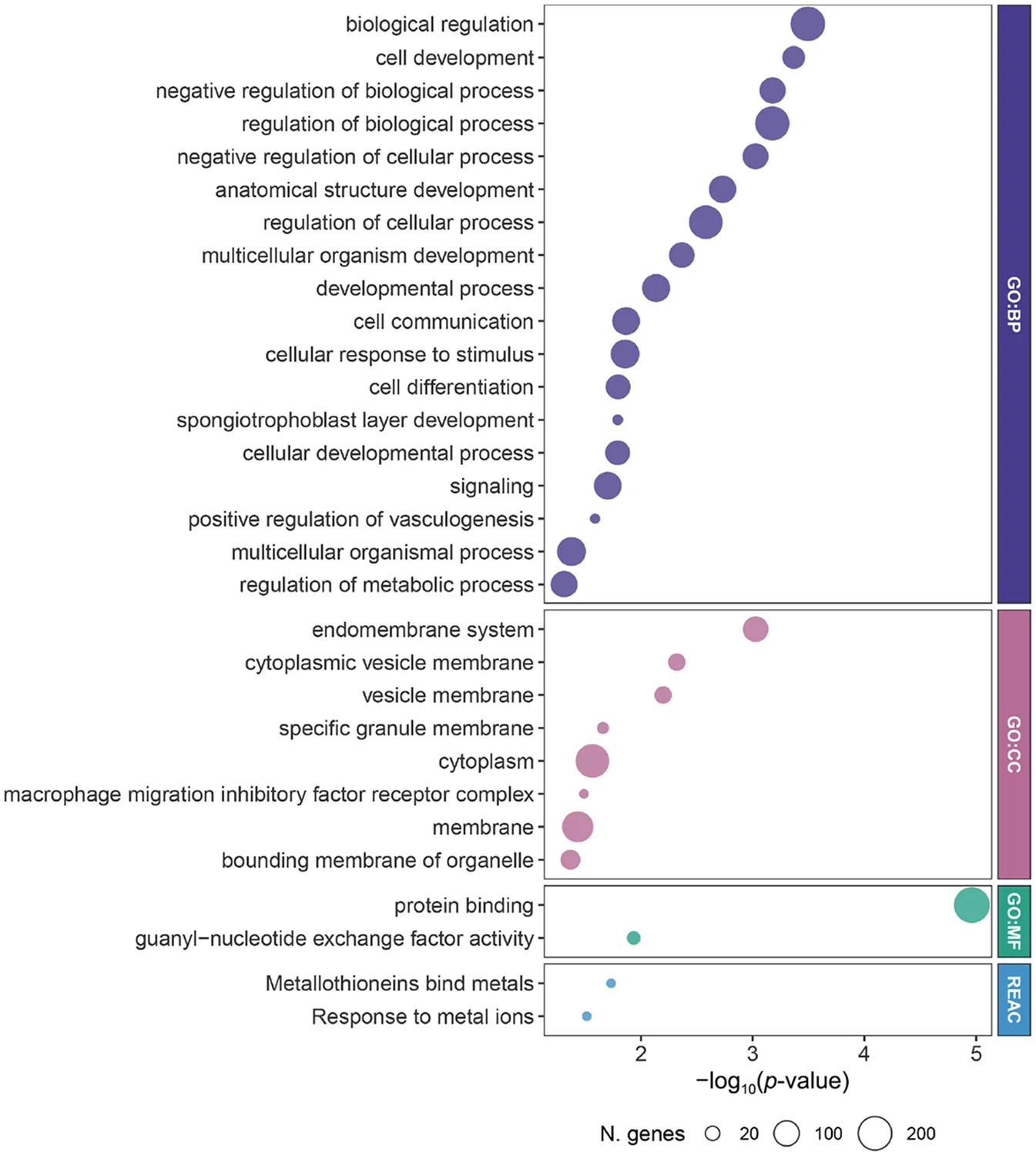
Pathway enrinchemnt enrichment analysis of DMP-associated genes. Bubble plots display the top enriched GO biological process (GO:BP), cellular components (GO:CC), molecular functions (GO:MF), and REACTOME (REACT) terms derived from DMP-annotated genes. The *x*-axis represents the enrichment significance (−log_10_[*p*-value]). Bubble size indicates the number of genes contributing to each term.

We performed a functional enrichment analysis of 396 genes associated with 346 DMPs identified in our EWAS at an FDR-corrected *p*-value < 0.10 (Supplementary Table S2). We retained pathways that reached an Bonferroni-corrected threshold of 0.05. This approached identified 18 GO biological processes, 8 cellular components, 2 molecular functions, and 2 REACTOME pathways with an SGA-related epigenetic signature (Fig. 2).

Of note, our analysis highlighted pathways related to early embryonic development, including "spongiotrophoblast layer development" (GO:0060712, adjusted *p* = 0.016), "positive regulation of vasculogenesis" (GO:2001214, adjusted *p* = 0.026), and "anatomical structure development" (GO:0048856, adjusted *p* = 0.002). Among the genes related to these processes, *ADM* emerged as key regulator participating in both the "spongiotrophoblast layer development" and "positive regulation of vasculogenesis" pathways (Supplementary Table S3).

## Discussion

Our study revealed a maternal epigenetic signature associated with SGA delivery, underscoring the potential role of DNA methylation in placental function and fetal development. We identified 14 CpG sites differentially methylated in association with the maternal risk of SGA delivery, including loci annotated to *GUCY2EP*, *MYOM2*, *NABP2*, *RNF41*, *UGT2A3*, *SHOC1*, *KCNK9*, *LOC105370276*, *GSE1*, and *DENND1A.* Functional enrichment analysis revealed the involvement of key pathways in early embryonic development and placental function, including "spongiotrophoblast layer development", "positive regulation of vasculogenesis", "anatomical structure development", "cellular developmental process", "multicellular organismal process", and "regulation of metabolic process". Among these, the spongiotrophoblast-like (intermediate) layer plays a particularly critical role in normal placental and fetal development, functioning as a structural, endocrine, and regulatory interface between maternal and fetal tissues^[23]^. A recent study reported that the development of the human intermediate layer begins around Weeks 4–5 post-conception, expands and functions throughout the second trimester, and persists at the implantation site until delivery^[24]^. Alterations of this layer or impaired placenta vascular development may lead to fetal growth restriction, thereby increasing the risk of SGA^[25]^. Normal placental function also requires proper formation, growth, and differentiation, processes that involve the biological functions of anatomical structure development and cellular/multicellular developmental processes^[26]^. To sustain fetal development, the placenta depends heavily on metabolic processes such as nutrient transport, hormore synthesis, and gas exchange^[27]^. Disruption of any of these pathways can result in placenta dysfunction and adverse pregnancy outcomes. Additionally, our data reveal that a higher proportion of SGA deliveries were observed in our study among mothers with a greater history of smoking during pregnancy and among those delivering female infants.

We identified DMP-associated genes that have not been previously associated with SGA, nor studied specifically in Black or nulliparous women. These genes are associated with placental development, vascular remodeling, and fetal growth regulation, indicating their potential contribution to the biological mechanisms leading to SGA. For example, the overexpression of *GSE1*, encoding for Genetic Suppressor Element 1, has been associated with increased tumor lymph node metastasis, as it promotes trastuzumab resistance in HER2-positive gastric cancer cells, indicating its role in cellular proliferation and survival pathways^[28]^. Animal models have also demonstrated that *Gse1* plays a pivotal role in placental development in mice^[29]^. Deletion of *Gse1* in the placental tissue impairs nutrient exchange, leading to placental dysfunction and fetal growth restriction. Together, these findings imply that dysregulation of *GSE1*'s expression through altered DNA methylation may impact placental growth and function, contributing to the risk of SGA.

Notably, two of the DMPs (cg12076885 and cg12076876) are located in the first exon of *MYOM2*, within or in close proximity to a cCRE with a proximal enhancer-like signature. *MYOM2* encodes for a structural protein localized in cardiac and fast skeletal muscle, anchoring myosin and titin filaments to maintain sarcomeres' integrity and normal muscle contraction^[30]^. Variants in the gene have been linked to pathological hypertrophic cardiomyopathy and the tetralogy of Fallot in animal models^[31]^. Although no prior studies have examined *MYOM2* variations in pregnancy outcomes, reduced plasma *MYOM2* levels have been identified as a biomarker for Duchenne muscular dystrophy (DMD), a condition that may be associated with impaired placental perfusion^[31,32]^. The identification of multiple CpG sites within a cCRE suggests a potential regulatory mechanism of *MYOM2* expression that is relevant to placental or fetal muscle development in SGA pregnancies.

Additionally, several DMP-related genes point toward biological processes that could plausibly influence fetal growth. For example, differential methylation in *NABP2*, a gene involved in DNA repair and cellular stress responses, may reflect reduced placental capacity to withstand oxidative or hypoxic insult, potentially compromising trophoblast function and fetal growth^[33,34]^. Epigenetic changes targeting *UGT2A3*, part of the placental detoxification and hormone metabolism machinery, may similarly affect the intrauterine environment during critical windows of development, thereby influencing fetal development^[35,36]^. *SHOC1* encodes a key protein for meiotic recombination in both males and females. Biallelic loss-of-function mutations of the gene lead to meiotic defects, maturation arrest, and infertility^[37]^. Its role in meiosis and chromosomal segregation suggests that dysregulation of *SHOC1* in the oocytes could affect embryonic chromosomal stability, placental implantation, and ultimately fetal growth. Finally, epigenetic modulations in *KCNK9*, an imprinted gene implicated in energy regulation and growth-related syndromes, may contribute to impaired placental or fetal development pathways that underlie growth restriction^[38]^.

Together, these patterns suggest that the epigenetic alterations we observed interrupt processes that are essential for healthy placental formation, metabolic regulation, and early embryonic development, which are core pathways central to the pathophysiology of SGA.

Although a few studies have specifically examined DNA methylation in relation to SGA risk among pregnant Black women, prior epigenetic studies in SGA have primarily focused on the candidate genes involved in hypoxia signaling, angiogenesis, and metabolic or imprinted growth regulation, including *EPO*, *H1F1A*, *VEGFA*, *LEP*, *PHLDA2*, *DHCR24*, *IGF2*, *GNASAS*, and *INSIGF*^[39,40]^. In contrast, none of the loci identified in our study directly overlap with these previously reported candidate genes, suggesting limited gene-level replication. However, functional annotation indicates potential convergence at the pathway level. Our identified genes are involved in developmental processes, cellular developmental processes, and regulation of metabolic processes, which may intersect with the biological processes implicated in hypoxia adaptation, placental function, and fetal growth. Taken together, although our findings do not replicate previously reported candidate loci, they support the notion that SGA-related epigenetic variation may act through broader regulatory networks beyond the canonical hypoxia and growth factor pathways identified in earlier targeted studies. Methodologically, prior studies have primarily used targeted approaches (e.g., polymerase chain reaction-based methods) to assess preselected DNA methylation markers, often across diverse tissue types such as placenta, cord blood, or cell-based models^[39-42]^. In contrast, our study did not focus on preselected candidates but instead provides a more comprehensive assessment of epigenomic variation using maternal blood samples. Though this approach enables broader discovery, it may not fully capture tissue-specific epigenetic changes. Our cohort also includes a wider maternal age range (≥ 13 years) and a relatively larger sample size, particularly for SGA cases, compared with some previous studies. Importantly, our study is among the few to specifically examine the epigenetic changes of SGA within a Black population. These strengths allow our findings to offer a more comprehensive characterization of epigenetic variation within this population. However, several limitations should be acknowledged, including the sample type constraints, the fact that assessments were limited to the first trimester, and the lack of any longitudinal follow-up of child-related outcomes.

We conducted an EWAS of whole blood from pregnant Black women during the first trimester, a critical window for placental and fetal development. Therefore, the maternal epigenetic alterations that we identified may serve as early biomarkers for SGA risk among Black women. Early identification of such epigenetic signatures could support the development of predictive tools to identify high-risk pregnant women and guide targeted monitoring or intervention strategies aimed at optimizing fetal growth and improving maternal health. These findings highlight the importance of integrating epigenetic biomarkers into precision maternal–fetal medicine to improve pregnancy outcomes. Black women were studied exclusively in our study because they face a disproportionately high risk of SGA delivery compared with other groups. The higher rates of SGA observed among Black women are likely associated with differences in exposure to chronic maternal stress, lower socioeconomic status, disparities in access to prenatal care, and a lack of insurance coverage, all of which may shape epigenetic characteristics. This disparity is driven by the cumulative effects of structural and interpersonal racism, which may be reflected in epigenetic variations.

Further investigations in pregnant Black women, as well as multiethnic cohorts, are needed to replicate and validate these findings. Future studies should incorporate longitudinal sampling across pregnancy and evaluate how the expression of genes highlighted by these epigenetic changes evolves over time. Integrating maternal methylation data with environmental exposures and adverse social determinants of health factors may also improve our understanding of how these factors jointly contribute to SGA risk. Ultimately, the development of predictive models combining epigenetic, clinical, and demographic biomarkers/risk factors could support early identification of pregnancies at risk and inform prenatal interventions.

Several strengths of this study merit consideration. A major strength of this study lies in the use of the nuMoM2b cohort, a large, well-characterized population specifically designed to investigate pregnancy outcomes among nulliparous women. Our analytic sample included 931 Black participants, representing one of the largest epigenetic cohorts of pregnant Black women studied to date^[43,44]^. The standardized recruitment protocol across eight academic medical centers and the longitudinal follow-up through all trimesters ensured consistent data collection and minimized selection bias. Moreover, the inclusion of participants enrolled early in pregnancy (6–13 weeks of gestation), a critical window of time for intrauterine development, strengthens the study's ability to capture early epigenetic changes potentially involved in fetal growth outcomes.

While our study revealed an intriguing epigenetic signature associated with SGA, several limitations should be acknowledged. The CpGs included in the Illumina Infinium MethylationEPIC assay represent only the 3% of the CpG sites in the human genome^[45]^. We did not account for other potential modulators of DNA methylation status, including hormonal levels (such as estrogen, progesterone, and cortisol)^[46-48]^, alcohol intake, diet, and comorbidities. The functional consequences of hyper- and hypomethylation at the identified CpG sites cannot be directly inferred, as the relationship between DNA methylation and gene expression is highly dependent on the genomic context and regulatory features. EWASes are subject to inflated test statistic distribution, which, if not properly addressed, may lead to false positive and false negative associations. Additionally, study site information was not available and therefore could not be included as a covariate. We acknowledge that residual site-specific variation, particularly given the environmental sensitivity of epigenetic modifications, may influence DNA methylation profiles. Although we adjusted the data for multiple sources of variability, including unaccounted confounding, individual-level covariates, cell type composition, and batch effects, our data showed moderate inflation (lambda = 1.32). Moreover, epigenetic variation may partially reflect underlying demographic and environmental differences, and future studies with larger and more diverse populations are needed to further disentangle these relationships.

## Conclusions

Our EWAS identified maternal epigenetic alternations at novel loci and implicates biological pathways involved in early embryonic development and placental function, providing new insight into the mechanisms that may contribute to SGA risk among pregnant Black women. Collectively, these findings highlight the potential of first-trimester DNA methylation profiles as an early biomarker for identifying pregnancies at increased risk of SGA delivery and lay the groundwork for developing precision prenatal screening and intervention strategies.

## Supplementary Material

Supplementary Table 3

Supplementary Table 1

Supplementary Table 2

**Supplementary information** This accompanies this paper online at: https://doi.org/10.48130/epi-0026-0006.

## Figures and Tables

**Table 1.** Demographic characteristics of pregnant Black participants.

	Total (*n* = 931)	SGA (*n* = 133)	AGA (*n* = 798)	*p*-value
Maternal Age				0.201^[1]^
Mean (SD)	23.46 (5.33)	22.85 (4.87)	23.56 (5.40)	
Smoked during pregnancy				0.017^[2]^
No	832 (100.0%)	111 (13.3%)	721 (86.7%)	
Yes	99 (100.0%)	22 (22.2%)	77 (77.8%)	
Education				0.069^[2]^
High school or less	397 (100.0%)	59 (14.9%)	338 (85.1%)	
Some college or college degree	469 (100.0%)	71 (15.1%)	398 (84.9%)	
Education beyond college	65 (100.0%)	3 (4.6%)	62 (95.4%)	
BMI				0.129^[1]^
Mean (SD)	28.86 (7.90)	28.09 (8.18)	28.98 (7.85)	
Infants' sex				0.002^[2]^
Female	460 (100.0%)	82 (17.8%)	378 (82.2%)	
Male	471 (100.0%)	51 (10.8%)	420 (89.2%)	

Maternal age refers to the age at enrollment. College includes either college degree or some college experience. *p*-value refers to Wilcoxon's rank sum test (1) or Pearson's *χ*^2^ test (2). SGA, small for gestational age; AGA, average for gestational age; SD, standard deviation; BMI, body mass index.

**Table 2.** Differentially methylated CpGs associated with SGA.

CpG ID	Position	Gene Symbol	Δ*β*	*p*-value	FDR *p*-value
cg20911180	chr11: 76,700,751	*GUCY2EP*	0.010	4.94E-08	0.021
cg00840694	chr8: 2,046,459	*MYOM2*	−0.016	6.59E-08	0.021
cg10928034	chr12: 56,224,331	*NABP2, RNF41*	0.002	7.85E-08	0.021
cg10406879	chr6: 165,091,497		0.010	1.54E-07	0.031
cg15878685	chr4: 68,953,138	*UGT2A3*	0.008	1.97E-07	0.032
cg13665149	chr9: 111,794,787	*SHOC1*	−0.005	2.69E-07	0.036
cg12669161	chr6: 3,170,598		−0.004	3.09E-07	0.036
cg01698714	chr6: 6,678,738		−0.005	3.89E-07	0.038
cg12076876	chr8: 2 046,264	*MYOM2*	−0.024	4.20E-07	0.038
cg24020826	chr8: 139,617,937	*KCNK9*	−0.007	4.64E-07	0.038
cg14273229	chr13: 80,362,139	*LOC105370276*	−0.010	5.71E-07	0.039
cg09396032	chr16: 85,635,856	*GSE1*	0.008	6.16E-07	0.039
cg14221454	chr9: 123,584,783	*DENND1A*	−0.004	6.27E-07	0.039
cg01168757	chr6: 135,173,257		−0.006	7.71E-07	0.045

Genomic positions are based on the GRCh38/hg38 human reference genome assembly. Δ*β*, delta beta; FDR, false discovery rate.

## Data Availability

NuMoM2b phenotype data are publicly available through the Eunice Kennedy Shriver National Institute of Child Health and Human Development Data and Specimen Hub NICHD (DASH, https://dash.nichd.nih.gov). Epigenomic data are publicly available through the NIH Database of Genotypes and Phenotypes (dbGaP, https://dbgap.ncbi.nlm.nih.gov/home) repository under accession number phs003992.v1.p1. Both are controlled access repositories.
